# Attitudes and beliefs of immigrants regarding HIV and AIDS in Mopani district, South Africa

**DOI:** 10.1080/17290376.2020.1831582

**Published:** 2020-11-10

**Authors:** Lunic B. Khoza, Hilda N. Shilubane, Mygirl P. Lowane

**Affiliations:** a Department of Advanced Nursing Science, University of Venda, Thohoyandou, South Africa; b Department of Public Health, Sefako Makgato Health Sciences University, Pretoria, South Africa

**Keywords:** Attitudes, beliefs, HIV/AIDS, immigrants

## Abstract

Sub-Saharan Africa faces and is severely affected by many conflicts. Human Immunodeficiency Virus (HIV) and Acquired Immunodeficiency Syndrome (AIDS) threaten both the physical and financial well-being of individuals in these struggling countries. This research aims to investigate the immigrants’ attitudes and beliefs regarding HIV/AIDS in the Mopani district, Limpopo Province. Qualitative and quantitative designs were used, and 200 immigrants were sampled. Data was collected using a questionnaire with closed and open-ended questions. Ethical standards were maintained. The study revealed that many respondents expressed discriminatory practices towards individuals infected by HIV. Many viewed promiscuity and the disease called Makhume (meaning illness caused by the omission of purification rites following the death of a family member) as causes of HIV/AIDS. These attitudes could hinder the achievement of healthy lives and the promotion of well-being at all ages if not addressed appropriately. The collaboration of various departments in the Mopani district is required to change these negative attitudes and beliefs that influence immigrants’ behaviours. Also, the use of audio-visuals and peer teaching is most successful in changing attitudes and beliefs.

## Introduction

Globally a marked diversity in HIV epidemiological patterns and trends has been reported to increase risks and vulnerability among immigrants (Grieb, Desir, Flores-Miller, Zelaya, & Page, 2017; Okoro & Whitson, 2017). Similarly, Sub-Saharan Africa has many challenges and is severely affected by many conflicts such as barriers to HIV education, reluctance to be tested for HIV, lack of knowledge concerning transmission and barriers to accessing health care among immigrants. Grieb et al. (2017) state that stigma is a powerful discrediting social label that perpetuates how individuals negatively view themselves in the communities. The authors further assert that understanding and combating the stigma surrounding HIV in the community is crucial to improving prevention and outcomes. The HIV/AIDS epidemic threatens both the physical and financial well-being of individuals in struggling countries. Inadequate levels of knowledge regarding HIV/AIDS is associated with the respondents’ negative attitudes towards people with HIV, and the use of condoms and is evident in their discriminative behaviour towards HIV positive individuals (Kouta, Phellas, & Charis, 2013; Nkwinika, Khoza, Lebese, & Shilubane, 2014).

Research indicates that negative attitudes towards HIV/AIDS, as well as persons infected with the virus, persist despite the increased knowledge of prevention (Kouta et al., 2013). These studies also found that a positive attitude is associated with safe practice. A study in Cyprus by Kouta et al. (2013) demonstrated that respondents reported avoiding contact with HIV-infected people as a way of preventing the infection. This research also revealed a lack of knowledge among respondents contributed to the isolation of those living with HIV. Furthermore, HIV-related stigma among Spanish-speaking Latinos in Baltimore, was found to be a barrier to care (Grieb et al., 2017).

Research conducted among refugee population established that fear was the typical reaction of respondents who were afraid of HIV/AIDS and used the word evil or killer to describe it. This fear could be caused by the lack of knowledge of the signs and symptoms as they could not identify those with AIDS until after they passed on. The study also found that respondents had both good and bad opinions about caring for HIV-infected family members. Respondents believed that people infected with the virus should keep their status confidential (Munene, 2012; Oliphant & Donaldson, 2019). Similarly, some studies indicated that inadequate HIV knowledge and the association of HIV with immoral behaviour creates fear of death as well as rejection by family members and the community (Andrew, Bhuiyan, Sung, Mawson, & Shahbazi, 2020; Grieb, Shah, Flores-Miller, & Page, 2015).

Although health education by peers has shown to increase knowledge, attitudes and practices related to AIDS in developing countries, negative attitudes and beliefs still prevail among misplaced individuals (Woodward et al., 2011). There is little knowledge about the attitudes and beliefs regarding HIV/AIDS among immigrants. Therefore, the primary aim of the current study was to investigate the immigrants’ attitudes and beliefs regarding HIV/AIDS.

## Methodology

### Study design

The study used both qualitative and quantitative designs. The approach enabled the researchers to analyse data using numerical information with statistical software to describe the results from the completed questionnaires (Creswell & Creswell, 2018). The respondents were conveniently sampled.

### Population, sample and sampling

The population of this study was those immigrants who reside in the Mopani district of the Limpopo Province. The study used non-probability sampling methods to purposively select one informal settlement with large numbers of immigrants receiving health care in a particular clinic, while a convenience sampling method was used to select 200 immigrants (Polit & Beck, 2017). Respondents were eligible for inclusion if they were over 18 years of age, self-identified as immigrants residing in the Limpopo Province and furthermore, only those immigrants who spoke Xitsonga and consented to participate formed part of the study.

### Data collection

The data was collected using questionnaires comprising of closed and open-ended questions to determine the respondents’ attitude and beliefs towards HIV/AIDS. Respondents gave their informed consent before taking part in the study. A first language Xitsonga-speaking expert translated the instrument developed initially in English into Xitsonga. Pre-testing of the questionnaire was done among ten immigrants with similar characteristics as the study respondents to ensure construct and face validity. Some questions were altered following the pre-test. The questionnaire was administered to participants who visited the clinic for consultation over ten weeks. A cubicle was used to maintain privacy when completing the questionnaires. The researchers filled in the responses of the respondents who could not read and write as they provided verbal answers. The pre-testing results were not included in the final analysis.

### Data analysis

Data was coded and analysed using a software programme called Statistic Package for Social Science (SPSS) version 23 to obtain frequencies and percentages. The open-ended questions were analysed qualitatively guided by the study questions. Initial classification of data was undertaken according to the questions to which respondents were responding. This was intended to strengthen and augment the findings of specific responses obtained from the closed-ended questions.

### Ethics

Ethical requirements aim to minimise the possibility of exploitation by ensuring that research respondents were treated with respect while they contribute to social good. The Department of Health in the Limpopo Province gave permission to conduct the study. It was explained to the respondents that participation was voluntary and their responses would remain anonymous. After explaining the purpose, risks and the benefits of the study, all respondents gave consent before completing the questionnaire and were assured that they could refrain or withdraw from participation without any penalty.

## Results

A total of 200 questionnaires were completed, and the results presented as biographical information, attitudes towards a person with HIV/AIDS and beliefs regarding HIV/AIDS.

## Biographical data

Most of the respondents, 112 (57%) were less than 30 years of age, while 88 (43%) were 33 years and older. Eighty-six (43%) were Mozambicans, 55 (28%) were Zimbabweans, and 59 (29.0%) consisted of other nationalities. There were more females 61% (122) than males 39% (78).

## Immigrants’ beliefs regarding HIV and AIDS

Table 1.

**Table 1.** Immigrants’ beliefs regarding HIV and AIDS.

	True		False		Do not know	
	n	%	n	%	n	%
1. Casual sexual relationships is the cause for AIDS	187	93.5	13	6.6	–	–
2. Married women have extremely low chances of contracting HIV compared to single women	192	96	8	4	–	–
3. Bathing after sex will help protect you against the virus	174	87	23	11.5	4	2
4. A person who appears healthy can have HIV	48	24	133	66.5	19	9.5

## Discussion

In the current study, there were more females (61%) than males (39%). Studies show that women are more likely to access health facilities as compared to men (Wang, Hunt, Nazareth, Freemantle, & Petersen, 2013). The study by Haroun et al. (2016) conducted in the United Arab Emirates to assess knowledge and attitudes to HIV/AIDS among University students revealed the majority of women (82%) than males (18%), contrary to the study by Grieb et al. (2017) which found that the gender difference was not alarming, for instance, it was 45% and 55% of males and females respectively. Studies show that in early adulthood and mid-life, women are more likely to access health facilities as compared to men. The women’s higher rates of using health services may be attributed to consulting for reproductive health (Wang et al., 2013).

Regarding the beliefs of HIV/AIDS, the majority of the respondents (94%) indicated that they believed that casual sexual relationships were the cause for AIDS while 6% (13) indicated that these relationships were not the only cause of the AIDS epidemic. The following descriptive open-ended question was included in the closed-ended questions: ‘why is casual sexual relationship/s the cause for AIDS?’ Respondents asserted that only people with multiple sexual partners get HIV, and married people do not get the virus. This is in line with Rodrigues, Prada, and Lopes (2019) who demonstrated that monogamy was perceived as assuring protection against STIs. In third world countries, the mode of spread for HIV is mostly through heterosexual intercourse, and most of such transmissions are through casual sexual relationships or sex before marriage (Robertson et al., 2012). This belief may give immigrants ‘a false sense of security while engaging’ in risky behaviours, as well as promoting discrimination and stigmatisation in that all HIV positive persons may be labelled as promiscuous (Oliphant & Donaldson, 2019; Robertson et al., 2012). Also, Mbonu, Van den Borne, and De Vries (2010) demonstrated that once a woman’s HIV status is positive, it is linked to casual sexual relationships unlike that of her male partner that is associated with transmission at the hair salon.

The response to the question *‘*Married women have low chances of contracting HIV compared to single women’ specified that 96% of the respondents stated that married women had low chances of contracting HIV compared to single women, and 4% (8) indicated that married women would not contract HIV. The finding could imply that the respondents had strong beliefs in fidelity maintained by married women. The respondents did not believe those male partners could infect their counterparts after contracting the disease outside of marriage since gender norms such as gender inequality encourage high-risk behaviours that increase males’ vulnerability to HIV (UNAIDS, 2012). According to HIV/AIDS advocacy strategies, faithfulness is one of the risk-taking activities in marriage (UNISEF, 2000). Married women who practice unprotected sex may be faithful to their husbands, and this puts them at risk of contracting HIV from their unfaithful partners (Rodrigues et al., 2019). Literature affirms that women are expected to be submissive to their male partners; as a result, they do not have control over their sex life. Furthermore, there is an assumption that once a woman enters into a contract of marriage, the husband has the right to unlimited sexual access to his wife (Jewkes, Dunkle, Nduna, & Shai, 2010; Rodrigues et al., 2019). Similarly, the study by Okoro and Whitson (2017) found that culturally, women were expected to be subordinate to men and were dictated by customs accordingly. The authors further indicated that women were not empowered to deal with issues of sexual abuse, and therefore, they are unwilling to discuss the experiences with others. A woman talking about sex is generally frowned upon and considered indicative of promiscuity (Okoro & Whitson, 2017).

The overwhelming majority of respondents 87% (174) mentioned that having a shower/bath immediately after sexual intercourse prevents HIV; however, 11.5% (23) did not agree with the statement while 2% (4) were unsure. This misconception may put immigrants at risk of contracting the virus through unprotected sex in the belief that bathing after sexual intercourse eliminates the virus. There is much to be done to remove the misconception, perhaps other strategies other than empowering immigrants with knowledge may help remove the misconceptions surrounding HIV/AIDS. The finding concurs with Pando et al. (2013), Alawad, Alturki, Aldoghayyim, Alrobaee, and Alsoghair (2019) and Dick, Ogbebor, and Azodo (2019) who demonstrated in their studies that respondents believed that bathing genitals, taking antibiotic prophylaxis and withdrawing the penis before ejaculation decrease the transmission of HIV.

The majority of the respondents, 66.5% (133) mentioned that a person who appears healthy could not have HIV. They had this belief despite the awareness education conducted by the Department of Health in Limpopo province. The finding is supported by Tompkins, Smith, Jones, and Swindells (2006) and Admassu, Tesfaye, and Dadi (2019) who also established that respondents believed that a healthy-looking person could not have HIV. Respondents could engage in unprotected sexual intercourse with infected partners, thinking that they do not have the disease because they look healthy. Furthermore, this mistaken belief could prevent individuals from using protection (Pando et al., 2013).

The outcome revealed the negative attitudes of immigrants towards a person with HIV/AIDS. There was a consensus among respondents that discriminatory practice in the community towards individuals infected by HIV was unbearable. In fact, respondents expressed fear of the disease and mixed feelings about caring for their HIV-infected relatives. Immigrants were afraid of getting HIV from an HIV-infected family member through mere touch and blood contact. One of them said: ‘someone with a cut is likely to get the virus through contact with an HIV-infected blood’. Some respondents did not trust the medical profession concerning the spread of HIV/AIDS. According to them, no one knows how HIV spread. A concern from a third participant was that they should be informed of the person’s HIV status in order to protect themselves and to purchase funeral policies for them**.** The majority stated that HIV or AIDS was ‘the disease caused by casual sexual relationships and was called Makhume’ (the illness caused by the omission of purification rites following the death of a family member)*.* This finding revealed one of the myths surrounding HIV/AIDS and the negative attitude of immigrants towards HIV positive individuals. The study conducted by Austin, Guy, Lee-Jones, McGinn, and Schlecht (2008) on knowledge and attitude and practice for HIV prevention and HIV infection risk among Congolese immigrants in Tanzania demonstrated that this group was not willing to use protective measures during sexual intercourse. The reason was that they wanted many children as this would be an advantage to them in terms of the food supply. This behaviour as well as transactional sex increases the risk of getting the virus, thereby preventing the achievement of healthy lives and the promotion of well-being for all ages (Bermudez et al., 2019).

A high number of respondents demonstrated negative attitudes and beliefs regarding HIV/AIDS and displayed inadequate knowledge of HIV infection, which could increase the risk for disease acquisition (Alawad et al., 2019; Macleod-Bluver, 2009). The negative attitudes could be caused by socioeconomic statuses, like poverty, low status of women, stigma and lack of knowledge (Alawad et al., 2019; Perkins, Voisin, & Stennis, 2013).

## Limitations

This study was conducted in one district of the Limpopo Province. The small sample number of immigrants hampers the results of the study from being generalised to all immigrants in South Africa.

## Implications, strength and weaknesses

South Africa has well-recognised strategies to fight HIV/AIDS epidemic such as community-based HIV awareness programmes and education, campaigns, research on HIV prevention and the introduction of anti-retroviral therapy (Alwafi et al., 2018). However, for various ethical and political reasons, it can be difficult for immigrants adjusting to a new society to agree with cultural and religious discrimination (Leung, Chin, & Petrescu-Prahova, 2016). The current study findings reveal that immigrants need to be empowered to deal with sociocultural barriers that are firmly entrenched and influence their behaviours, ignorance on sexual health and the need to develop ‘destigmatise HIV’ strategies. The lack of sexual health literacy by African immigrant women was also a key finding in the studies conducted by Okoro and Whitson (2017). The authors, therefore, recommended that sexual and reproductive health education needs to be both culturally responsive and at an appropriate literacy level (Okoro & Whitson, 2017). The current study implies that there is a need to change the mind-set of the immigrants towards those living with HIV/AIDS in Limpopo. Similarly, this perception was described in the study by Alwafi et al. (2018) in Saudi Arabia in that nearly half of the respondents indicated that people living with HIV/AIDS should be isolated.

## Recommendations

The following suggestions are recommended to cultivate positive attitudes and address the beliefs held by immigrants regarding HIV/AIDS in the Mopani district. Qualitative studies are required, and the findings used to develop programmes tailored to the unique needs of the immigrants. Furthermore, the development of educational materials in the language understood by most immigrants is essential. The use of audio-visuals and peer teaching is most successful in changing attitudes when compared with once-off educational programmes. Furthermore, there must be collaboration between health care workers and other stakeholders in eliminating negative attitudes and beliefs of immigrants towards individuals infected with HIV and those who have AIDS.

## Conclusion

The current study revealed some beliefs and negative attitudes held by immigrants towards HIV/AIDS as well as infected individuals. These negative attitudes need addressing if the country seeks to bring down the infection rate, which could be fuelled by non-disclosure by people living with HIV due to fear of stigma. The collaboration of various departments in the Mopani district is required to change these negative attitudes and beliefs influencing immigrants’ behaviour. Rather than targeting this high-risk group in the context of contracting HIV, the information outlined in the current study demonstrates that achieving immigrant women’s empowerment on HIV/AIDS is an urgent issue. The public health services in Limpopo should consider introducing specific HIV positive interventions that will reduce harmful traditional and cultural practices.
